# Th17/Treg Ratio and Disease Activity in Systemic Lupus Erythematosus

**DOI:** 10.22088/cjim.10.1.65

**Published:** 2019

**Authors:** Yuliasih Yuliasih, Lita Diah Rahmawati, Rizki Maulidya Putri

**Affiliations:** 1 Department of Internal Medicine, Rheumatology Division, Faculty of Medicine, Airlangga University, Surabaya, Indonesia

**Keywords:** Systemic Lupus Erythematosus, disease activity, Th17, Treg, Th17/Treg ratio

## Abstract

**Background:**

Systemic lupus erythematosus (SLE) is an autoimmune disease that is characterized by T-cells imbalance. There are ongoing controversies about the role of specific T-helper cell subsets and their cytokines. The study aimed to confirm the disturbance of Th17/Treg ratio in SLE patients.

**Methods:**

Subjects were SLE patients who met the American College of Rheumatology 1997 criteria. Disease activity assessment was measured by SLAM index. Th17 and Treg level was measured by flow cytometry. Th17 level was evaluated as CD4^+^L17 whilst Treg as CD4^+^Foxp3^+^. Final result is stated as Th17/Treg ratio.

**Results:**

Thirty female subjects with active SLE had mean SLAM Score of 29.3±3.88, C3 level 25.2 (6-59.5), C4 level 15.25 (5-54.3), ESR 62.1±37.85, CRP 30.16±59.45, and anti-dsDNA 155.32±186.10. Higher Th17 level was found in SLE patients compared to healthy subjects (30.09 pg/ml vs 13.01pg/ml; 12.60% vs 0.91%). However, it did not correlate to disease activity (p>0.05; r=-0.28). Regarding Treg level, there was no significant difference between active SLE and healthy subjects (12.85 vs 11.05 pg/ml; 9.57% vs 2.05%). Treg level negatively correlated to SLE disease activity (p<0.01; r=-0.73). Th17/Treg ratio was 3.28±2.22% and it positively correlated to SLE disease activity (p<0.01; r=0.78).

**Conclusion:**

Th17/Treg ratio is positively correlated with disease activity. Th17 level is elevated but not correlated with disease activity. Decrease of Treg level is not significant though correlated with disease activity in SLE patients.

Systemic lupus erythematosus (SLE) is an autoimmune disease that is triggered by overly activated autoantigen-specific T cells and characterized by the presence of multiple autoantibodies in target organs (1, 2). These antibodies are end-results of the apoptotic program impairment and the decrease of apoptotic material clearance. The apoptotic materials, including the immunogenic modified histones in particular, are captured by antigen presenting cells (APC) then presented to T-cells. This reaction consequently leads to T-cells hyper-responsiveness and immune complex, causing inflammation and organ damage (3-5). New studies at cellular level have shown that homeostasis disturbance between Effector T-cells (Th) and Regulator, T-cells (Treg) contribute significantly to the pathogenesis of SLE. This new paradigm is slowly replacing the old one about the roles of B-cells and Th1/Th2 in SLE pathogenesis. There are many recent studies that have been reported that Th17 cells produce several pro-inflammatory cytokines, such as IL-17A, IL-17B, and IL-22, which can cause tissue injury and eventually organ damage. 

IL-17 level is abnormally high in active SLE patients. Thus, the higher IL-17 level found in SLE patient, more organ damage occurs (6-9). Treg stimulates IL-2 and TGF-β, which have protective effects in autoimmune disease. Treg function is interfered by IL-6. The elevation of IL-6 level in SLE converts Treg to Th17 (10). The mechanism of suppression by Treg is classified into 4 mode of actions: inhibitory cytokines, cytolysis, metabolic disruption, and targeting dendritic cells. Treg secretes inhibitory cytokines IL-10, IL-35, and TGF-β which directly inhibit effector T cells (Th) activity (11). The imbalance of immunogenic tolerance of dendritic cells limits the expansion of Treg and causes a lower Treg cells level. Both Treg level and function are imperative in pathogenesis and activity of SLE. 

Compared to Treg, Th17 levels are essentially higher in the early stage of SLE. This imbalance is deemed to be connected to the disease activity of SLE itself, which can be measured by a valid (0.9) and reliable (0.83) scoring system like Systemic Lupus Activity Measure (SLAM) index. SLAM index is applicable for SLE patients who have not undergone treatment using immunosuppressive agents (9, 12, 13). It is suitably used in this study, considering most of SLE patients in Dr. Soetomo General Hospital Surabaya are referred by secondary health care yet untreated. Our previous study reported that the mortality rate of SLE patients admitted to Dr. Soetomo General Hospital in 2004 was 22,9% (14). It was significantly higher than global mortality rate for SLE (0,025%) (15). Hence, early evaluation of Th17 and Treg level is very crucial in order to take immediate intervention, to improve prognosis, and to suppress mortality in SLE patients. This study aims to evaluate Th17 cells and Treg level and to confirm the association of Th17/Treg ratio with disease activity in patients with active SLE.

## Methods

**Subjects and Sampling Method: **This study was approved by the Ethics Committee of Dr. Soetomo General Hospital for human research number 155/Panke. KKE/III/2017 and informed consent was obtained from all participants. Subjects in this study were enrolled using *consecutive sampling* method. Sample size was determined by the following formula (16). 

The subjects were thirty female patients with active SLE who matched the revised American College of Rheumatology 1997 (ACR) SLE criteria and had an active disease characterized by Systemic Lupus Activity Measure (SLAM) index > 20. Based on a previous study, higher than 20 SLAM index indicates highly active SLE. These patients were hospitalized in the Internal Medicine ward, Dr. Soetomo General Hospital, Surabaya, Indonesia. All SLE patients in this study were females, considering the number of SLE case from Indonesian female population is 40 times higher than male population (14). Patients with infections, steroid or immunosuppressant medications, malignancies, history of smoking, acute coronary syndrome, tuberculosis, HIV/AIDS, and inflammatory bowel disease were excluded from this study**. **Four healthy subjects were also recruited and had their Th17 and Treg level tested as baseline. 

**SLAM Index Scoring: **SLAM index was introduced in 1988 by Liang et al. It consists of 31 items to evaluate clinical symptoms and laboratory findings and to review 11 system-organs using 1-3 scoring system. The total score depends on the severity of organ damage, ranging from 0 to 86 points. The higher the score implies more active disease. The sensitivity of SLAM index compared to SLEDAI/BILAG are identical, despite Petri et al. (2005) mentioned that SLEDAI is the most sensitive (2). However, considering the wide range of SLEDAI scoring system (1 to 8) which can easily cause scoring bias between different examiners, SLAM index is more suitable for this study as the scoring scale is only ranged from 1 to 3. 

**PBMCs Isolation: **In order to optimally analyze Th17 and Treg using flow cytometry, T-cells specimen was obtained by isolating mononuclear white blood cells (lymphocyte and monocyte) from peripheral blood. Peripheral blood mononuclear cells (PBMCs) are separated from 4 ml of heparinized venous blood by a density gradient centrifugation method using Ficoll Histopaque. 

**Stimulation of the cells: **Normal PBMCs were stimulated in media for 5 hours using PMA/Ionomycin (at 50 ng/ml and 1μg/ml respectively for 1 million cells) in the presence of BD GolgiStop™ protein transport inhibitor (Cat No. 554724). 4 μl of BD GolgiStop™ was added for every 6 ml of cell culture and mixed thoroughly. BD GolgiStop™ should not be kept in the culture for longer than 12 hours. 

**Staining of the cells: **Cells were collected *in vitro *using a protein transport inhibitor, centrifuged (250 x g) out of the medium containing BD GolgiStop™, and suspended in BD Pharmingen™ Stain Buffer (FBS); Cat No. 554656. Cells were counted and adjusted with concentration to 20 million cells/ml in stain buffer (FBS). Cells were fixated gently by re-suspending a pellet of 1 million cells in residual volume of wash buffer and adding 2ml of 1x human FoxP3 buffer A. Afterwards, cells were incubated for 10-20 minutes at RT in the dark and centrifuged 500 x g for 5 minutes, and then got the fixative removed. Cells were then washed and each pellet was re-suspended in 2ml of stain buffer (FBS), and centrifuged 500 x g for 5 minutes. Lastly, the buffer was removed and cells were stored or preceded to permeabilization of cells.

**Permeabilizing the fixed cells: **DMSO was removed from frozen cells by washing twice with 2 ml/tube of stain buffer (FBS) and centrifuged 500 x g for 5 minutes at RT, got the buffer removed and washed again. Cells were permeabilized gently by suspending the pellet in residual volume of buffer and then adding 0.5 ml of 1x working Human FoxP3 Buffer C solution to each tube.

 Next, cells were incubated for 30 minutes at RT protected from light and washed twice with 2 ml/tube of stain buffer (FBS), centrifuged 500 x g for 5 minutes at RT and got the buffer and stain removed.

**Staining with the cocktail: **Cells were thoroughly suspended and fixed/permeabilized in 50 μl -150 μl of stain buffer (FBS) and added 20 μl/test of cocktail or appropriate negative control. Next, cells were incubated at RT for 40 minutes in the dark and should be protected from light throughout staining and storage. Afterwards, cells were washed twice with 2 ml/tube of stain buffer (FBS), centrifuged 500 x g for 5 minutes at RT and got the buffer removed then analyzed.

**Flow cytometry analysis: **Flow cytometry analysis was performed to measure Th17 and Treg percentages using human Th17/Treg phenotyping kit (BD Biosciences, Texas, USA) and Becton & Dickinson Flow Cytometry. Both of these cells were measured from PBMCs of the SLE patients and controls. 

Cells were fixed and permeabilized with Fix/Perm buffer (BD Bioscience, USA). Afterwards, cells were labeled with PEanti-human IL-17A (BD Bioscience, USA) for Th17 identification and FITC anti-human CD4 antibody/PerCP anti-human FoxP3^+^ antibody (BD Bioscience, USA) for Treg identification. Flow cytometry was analyzed using BD CellQuest Pro software (BD Bioscience, USA).

**Statistical analysis: **All collected data were arranged in tabular form and processed statistically using the SPSS 21.0 program (IBM Corp., Armonk, NY, USA). Test of data normality was analyzed using Shapiro-Wilk normality test. The correlation between the Th17/Treg ratio and disease activity was analyzed using Pearson’s correlation coefficient test (for normal distribution) or Spearman’s rank correlation coefficient test (for abnormal distribution); A p<0,05 was considered statistically significant.

## Results

**Demography and clinical characteristics of the subjects: **Thirty female patients with active SLE had their SLAM index scored and resulted with 29.3±3.88. The mean age of SLE patients in this study was 31.3±10.5 years old. Most of subjects were anemic with serum hemoglobin level of 7.12±2.32. ESR level was at 62.1±37.85 and considered as high. Both C3 and C4 level were low, at 25.2 (6-59.5) and 15.25 (5-54.3) respectively. 

High level of anti-dsDNA was found at 155.32±186.1. The clinical information and the laboratory results of the subjects are shown in table 1. 

**Table 1:** Clinical information and laboratory results of the subjects

**Characteristics**	**Total (n = 30)**
Sex (Female/Male)	30 : 0
Age (years) (Mean ± SD)	31.3 ± 10.5
Hemoglobin (Mean ± SD)	7.12 ± 2.32
Lymphocyte (Mean ± SD)	967.87 ± 427.78
Thrombocyte (Mean ± SD)	212766.67 ± 206889.06
ESR (Mean ± SD)	62.10 ± 37.85
CRP (Mean ± SD)	30.16 ± 59.45
C3 (Median)	25.20 (6.00–59.50)
C4 (Median)	15.25 (5.00–54.30)
anti dsDNA (Mean ± SD)	155.32 ±186.10

Table 2 shows the distribution of clinical and laboratory manifestations according to ACR 1997 criteria. The most common manifestation was arthritis (76.7%). Renal disease and neurological disorder were diagnosed in 50% and 20% of the subjects, respectively. Hematological manifestations were found in all subjects: 66.7% with hemolytic anemia, 3.3% leukopenia, 90% lymphopenia, and 40% with thrombocytopenia. 

In this study, ANA test was found positive in 73.3% subjects and followed by 46.7% subjects with positive anti-dsDNA. 

**Table 2:** Clinical manifestations of the subjects according to ACR 1997 criteria

**ACR 1997 characteristics**	**Percentage (%)**	
Malar rash	13.3
Discoid rash	26.7
Photosensitivity	43.3
Oral ulcer	33.3
Arthritis	76.7
Serositis	46.7
Renal disease	50.0
Neurologic disorders	20.0
**Hematologic disorders**	
Hemolytic anemia with reticulosis Leukopenia < 4000/mm^3^ Lymphopenia < 1500/mm^3^ Thrombocytopenia < 100×10^3^/mm^3^	66.7 3.3 90.0 40.0
**Immunologic disorders**	
Anti-dsDNA positive Anti-Sm positive Anti-phospholipid positive	46.7 23.3 0
ANA test positive	73.3

**Figure 1:**
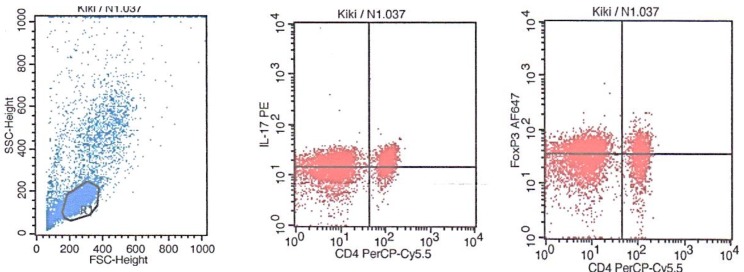
Flow cytometry analysis of CD4 expressing IL-17A and Foxp3

**Figure 2:**
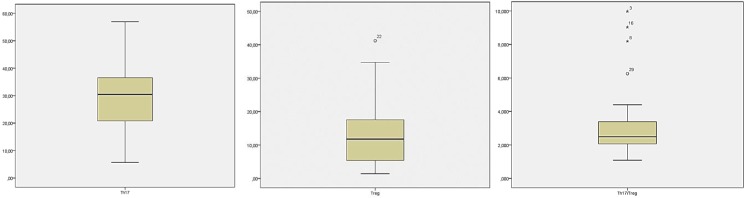
Th17 and Treg level measured from subjects’ peripheral blood mononuclear cells (PBMCs). A. Subjects’ Th17 mean level. B. Subjects’ Treg mean level. C. Subject’s Th17/Treg mean ratio

**Th17 and Treg level in patients with active SLE: **The measurement of Th17 and Treg levels was performed by analyzing PBMCs using flow cytometry. All dots captured in a form of scatter diagram by either *forward *or* side scatter* represent the whole leukocytes (granulocytes, lymphocytes, and monocytes). CD4 lymphocytes expressed both IL-17A and Foxp3 plotted within gating area (R1) which were then processed and analysed separately (Fig. 1). Th17 cells expressed CD4+ IL-17+, whereas the Treg cells expressed CD4+FoxP3+. Th17 level in active SLE patients was 30.09±12.60% (fig.2a) with baseline level of 13.01±0.91% in healthy subjects. Treg level was 12.85±9.57% (fig. 2b), which showed no difference compared to healthy subjects (11.05±2.05%). Additionally, we found that the subjects’ Th17/Treg ratio was also higher (3.82±2.22) compared to that of the healthy subjects’ (1.20±0.20) (fig. 2c). 

**Figure 3:**
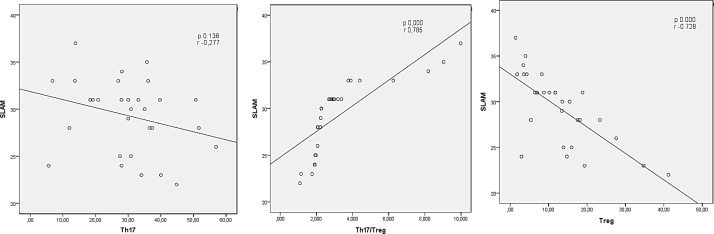
Correlation between Th17, Treg, and Th17/Treg ratio from subjects’ peripheral blood mononuclear cells (PBMCs) and disease activity. A. Th17 level vs. SLAM. B. Treg level vs. SLAM. C. Th17/Treg ratio vs. SLAM

**Th17 correlation to disease activity in patients with active SLE: **A correlation of Th17 cells to disease activity is shown in fig. 3a. Th17 serum level (CD4+IL-17) and SLAM score were analyzed by Pearson’s correlation test and the result shows that it has no correlation to SLE disease activity (r=-0.277, P=0.138). This study also analyzed Th17 level’s correlation to hemoglobin and lymphocyte (using Pearson’s correlation test) and to proteinuria (using Spearman’s correlation test). Th17 level was found to have a correlation with hemoglobin level (r=507, P=0.004). However, it does not correlate to lymphocyte count (r=-0.261, P=0.164) and proteinuria (r=-0.200, P=0.289)**.**

**Treg correlation to disease activity in patients with active SLE: **Spearman’s rank correlation test was used for correlation analysis between Treg serum level (CD4+Foxp3+) and SLAM score in total SLE patients. As shown in fig. 3b, Treg serum level has a strong inverse correlation to SLE disease activity (r=-0.728, P=0.000).

**Th17/Treg ratio correlation to disease activity in patients with active SLE: **Pearson’s correlation test was used for analysis between Th17/Treg ratio and SLAM score in each SLE patient. In fig.3c, correlation analysis shows that there is a strong correlation between Th17/Treg ratio with SLE disease activity (P=0.000, r=-0.728). This study also analyzed the correlation of Th17/Treg ratio to ESR, CRP, C3, C4, and anti-dsDNA, in which the correlations were found only for C3 (r=-0.69, p<0.01) and C4 (r=-0.52, P=0.52, p<0.01).

## Discussion

In this study, all subjects were productive young women who presented high SLE activity confirmed by SLAM index, complement level, ESR, and CRP. We expected lower Treg level and elevated Th17/Treg ratio occurs in active SLE patients. Our results showed that higher Th17 level, lower Treg level, and higher Th17/Treg ratio were found in SLE patients compared to healthy patients. It was consistent to previous studies involving active SLE subjects. Our results also confirmed that Treg level has an inverse correlation with disease activity measured by SLAM index, whilst Th17/Treg ratio shows a strong correlation to disease activity.

Elevated Th17 level was found in active SLE patients. According to past studies, Th17 and IL-17 have a significant role in SLE progression and are found higher in active SLE compared to inactive SLE (17, 18). We observed no such correlation exists between Th17 level and disease activity, despite several studies claimed for their linear correlation. The explanation to this finding is that IL-7 might correlate to disease severity instead of disease activity. Th17 are able to secrete tumor necrosis factor (TNF), IL-2, and IFN-γ (10, 19). Based on this theory, in patients with SLE (including new-onset disease), elevated Th17 and IL-17 levels increase their infiltration ability to target organs (6). High Th17 level in SLE patients results in excessive production of pro-inflammatory cytokines and chemokines, so that the systemic condition of SLE patients will always be in an inflammatory state, causing more severe inflammation and organ failures, especially renal and hematological impairments. The presence of kidney and hematological impairments is a sign of a severe lupus. Our previous study observed a high mortality rate caused by renal and hematological impairments in Dr. Soetomo General Hospital Surabaya (14). As for the non-correlating Th17 and proteinuria, it might be due to small sample size of this study.

Yang et al. found that Th17 level in SLE patient was correlated to disease activity and its level would increase during lupus flare and decrease after treatment (17). Zickert et al. (2015), Kwan et al., 2009, also reported that IL-17 was related to the activity of lupus nephritis. IL-17 was found abundantly in lupus nephritis (LN) patients, although molecular mechanism which explains T-cells or IL-17 dependent tissue damage of LN was not clearly elaborated. There are some findings that demonstrated the contribution of T-cell signaling pathways to the expression of LN (20). High IL-17 baseline can predict unfavorable histopathology response. IL-17 is also responsible in aplastic anemia (21).

Our study demonstrated that there was no substantial decrease of Treg level in SLE patients. Previous studies provided inconsistent evidence; some stated the decrease Treg level (22), others declared its similarity with healthy patients (23) or even elevated Treg level (24). Our results might be explained by other studies that mentioned the decreasing function of Treg in SLE without the decreased level in peripheral blood (25). This variant might be caused by different methods of isolation used in this study and subjects’ various characteristics, considering the lack of consensus for Treg phenotyping method in human (26). This study showed a slight elevation of Treg compared to healthy subjects. The elevation could be caused by mixed activated T-cells during the counting in which T-cells can adaptively convert to Treg (27). Another plausible explanation is that Treg might attempt to control the exacerbated immune response in active disease (28).

In association with disease activity in our study, Treg had a significant inverse correlation. These findings were in accordance to a study by Maraghy et al., (2016) in which Treg had a significant correlation with renal impairment and hematological impairment (28). Treg CD4+Foxp3 secretes an abundant amount of cytokines that proliferate CD4 T-cells specific towards nuclear antigen, such as nucleosome, which is known as pathogenic antibody in lupus nephritis (29). Antibody nucleosome level is higher in lupus with renal impairment. Decrease of Treg correlates with disease severity (30). A study by Shakweer et al., (2016) showed the elevated Foxp3+ expression in non-proliferative lupus nephritis compared to the proliferative one (31). CD4+Foxp3 without CD25 expression is an important subset from Foxp3+ T cells that has been found frequently in SLE patient. CD4+Foxp3 may be distinctive in SLE but the origin and function of this subset of Treg are mostly unknown (29).

Furthermore, our study found a correlation between Th17/Treg ratio and disease activity of SLE. This study confirmed that Th17/Treg ratio could be used to differentiate active and inactive SLE, as well as its severity (32). The greater ratio represents higher and more severe disease activity. We also analyzed the correlation between Th17/Treg ratio and certain laboratory parameters frequently used in daily practice to determine severity of disease, such as ESR, CRP, C3, C4, and anti-dsDNA. We observed that there was a significant inverse correlation between Th17/Treg ration with both C3 and C4. This finding successfully confirmed a study by Narayanan et al., (2011) and Bahlas & Damiati (2014) (33). 

According to Lam & Petri (2005), a measurement of C3 and C4 level is helpful for diagnosing SLE, although hypocomplementemia is not specific for SLE. On the other side, no correlation was found for ESR, CRP and anti-dsDNA levels. ESR measurement has many limitations and a low diagnostic value. It is only useful for treatment response evaluation (2). Cengic et al. (2002) mentioned that ESR and CRP levels are to evaluate the relapse of the disease and to distinguish infection (34). Most of the previous studies tried to correlate anti-dsDNA level to disease activity, but their findings showed inconsistencies (33, 35). The number of SLE patients with positive anti-dsDNA in our population is considerably low. Thus, it might affect the result of our study. All subjects in this study were under the Indonesian government’s health insurance. However, several examinations were not fully covered.

In conclusion, elevated Th17 level and insignificant decrease of Treg level, and Th17/Treg ratio correlate to disease activity and disease severity in patients with active SLE. Elevated Th17 level significantly contributes to chronic inflammation process in SLE. Imbalance of Th17/Treg ratio is an important determinant for SLE disease activity and disease severity.
